# FOXF1 mediates mesenchymal stem cell fusion-induced reprogramming of lung cancer cells

**DOI:** 10.18632/oncotarget.2413

**Published:** 2014-09-09

**Authors:** Hong-Jian Wei, Jac A. Nickoloff, Wei-Hong Chen, Hen-Yu Liu, Wen-Cheng Lo, Ya-Ting Chang, Pan-Chyr Yang, Cheng-Wen Wu, David F. Williams, Juri G. Gelovani, Win-Ping Deng

**Affiliations:** ^1^ Graduate Institute of Biomedical Materials and Engineering, College of Oral Medicine, Taipei Medical University, Taipei 110, Taiwan; ^2^ Stem Cell Research Center, Taipei Medical University, Taipei 110, Taiwan; ^3^ Department of Environmental and Radiological Health Sciences, Colorado State University, Fort Collins, CO 80523, USA; ^4^ Department of Neurosurgery, Taipei Medical University Hospital, Taipei 110, Taiwan; ^5^ School of Medicine, Taipei Medical University, Taipei 110, Taiwan; ^6^ Department of Internal Medicine, College of Medicine, National Taiwan University, Taipei 100, Taiwan; ^7^ Institute of Biochemistry and Molecular Biology, National Yang Ming University, Taipei 112, Taiwan; ^8^ Wake Forest Institute of Regenerative Medicine, Winston-Salem, NC 27157, USA; ^9^ Department of Biomedical Engineering, College of Engineering and School of Medicine, Wayne State University, Detroit, MI 48201, USA; ^10^ Center for Molecular Medicine and Genetics, Wayne State University, Detroit, MI 48201, USA

**Keywords:** cell fusion, mesenchymal stem cell, lung cancer cell, reprogramming, FOXF1, p21

## Abstract

Several reports suggest that malignant cells generate phenotypic diversity through fusion with various types of stromal cells within the tumor microenvironment. Mesenchymal stem cell (MSC) is one of the critical components in the tumor microenvironment and a promising fusogenic candidate, but the underlying functions of MSC fusion with malignant cell have not been fully examined. Here, we demonstrate that MSCs fuse spontaneously with lung cancer cells, and the latter is reprogrammed to slow growth and stem-like state. Transcriptome profiles reveal that lung cancer cells are reprogrammed to a more benign state upon MSC fusion. We further identified FOXF1 as a reprogramming mediator that contributes not only to the reprogramming toward stemness but also to the p21-regulated growth suppression in fusion progeny. Collectively, MSC fusion does not enhance the intrinsic malignancy of lung cancer cells. The anti-malignant effects of MSC fusion-induced reprogramming on lung cancer cells were accomplished by complementation of tumorigenic defects, including restoration of p21 function and normal terminal differentiation pathways as well as up-regulation of FOXF1, a putative tumor suppressor. Such fusion process raises the therapeutic potential that MSC fusion can be utilized to reverse cellular phenotypes in cancer.

## INTRODUCTION

Cell fusion is a complex and highly regulated process with critical roles in several physiological (fertilization, tissue regeneration) and pathophysiological (viral infection, cancer) events. Physiological cell fusion is essential for many cellular events such as fertilization, formation of placenta and muscle fibers, bone homeostasis, and immune response [1–2]. Cell fusion is also a strong inducer of aneuploidy and genomic instability in cancerous tumors. Aneuploidy is a remarkably common characteristic of human cancer and has been proposed to promote tumorigenesis, and/or to contribute to tumor progression. Cancer is rarely derived from mutational events unless genome stabilization pathways like DNA repair and checkpoints are defective [3]. Many types of malignant cells display promiscuous fusion among themselves or with normal cells to generate phenotypic heterogeneity [4]. Thus, cell fusion is thought to induce drastic genomic variation that contributes to tumor progression, including enhanced phenotypic diversity, drug resistance, and metastatic potential [5–6].

Various types of tumor microenvironment cell can fuse with malignant cells [7–10]. Such fusion progeny have been detected in numerous animal models and in human cancers [11–12]. Mesenchymal stem cell (MSC) is also an important fusogenic candidate in the tumor microenvironment. MSCs are multipotent cells that can self-renew and differentiate into various somatic lineages that contribute to the maintenance and regeneration of a variety of tissues, including bone, adipose tissue, cartilage and muscle [13]. We previously demonstrated that MSCs can migrate to and engraft into microscopic tumor lesions [14]. The tropism of MSCs to tumors has also been established for various types of cancer [15–19]. Upon homing to the tumor lesions, MSCs reveal many promoting and supporting effects on tumor progression, including immune response suppression [20], enhancement of tumor growth [21–22], metastasis [17, 23] and stroma development [14], and regulation of cancer stem cell population [24–25]. These findings suggested that MSCs play critical roles in the tumor microenvironment during tumor progression. The interaction between malignant cells and MSCs, including cell fusion, has emerged as a key issue that bears further examination.

Cellular reprogramming is the process in that a differentiated and specialized cell is conversed to different cellular state that would not occur under normal physiological conditions. Reprogramming can be achieved by various methods such as somatic cell nuclear transfer, cell-cell fusion and introduction of transcription factors [26]. Cell fusion is a nuclear reprogramming technique that involves fusing two or more cell types to form a single identity. The capacity of adult stem cells exhibiting phenotypic potentials beyond their original lineage is termed as stem cell plasticity, also known as transdifferentiation ability, which is important for stem cell-mediated regeneration [27]. Stem cells can reprogram the somatic cells via cell-fusion events, which recently have been implied to be involved in the transdifferentiation ability of adult stem cell [28–29]. Stem cell fusion with somatic cells can restore regenerative capacity of terminally differentiated cells and be applied for transplantation and cell therapy [30–32]; however, the biological output of stem cell fusion with malignant cells remains controversial. Several studies have shown that stem cell enhances malignant characteristics upon fusion with malignant cells [33–35], but some conflicting reports revealed that stem cell reduces tumorigenicity through cell fusion [36–38]. Although greatly advancing the field, it is unclear that upon MSC fusion cancer cells are more malignant if they gain self-renewal and migratory abilities or are more benign if their genetic or epigenetic defects are corrected. In this study, we intend to investigate the functional roles and mechanistic regulation of cellular fusion between MSCs and malignant cells. Upon fusion with MSC we showed that lung cancer cells have decreased tumorigenicity and conferred stem cell characteristics. Transcriptome profiles also revealed that lung cancer cells are reprogrammed to a more benign state through restoring the expression of FOXF1, a putative reprogramming mediator. Our results suggest that MSC fusion can reprogram lung cancer cells to a nontumorigenic, stem-like state instead of a more malignant state.

## RESULTS

### Spontaneous fusion of lung cancer cells and MSCs forms synkaryonic hybrids

**Figure 1:**
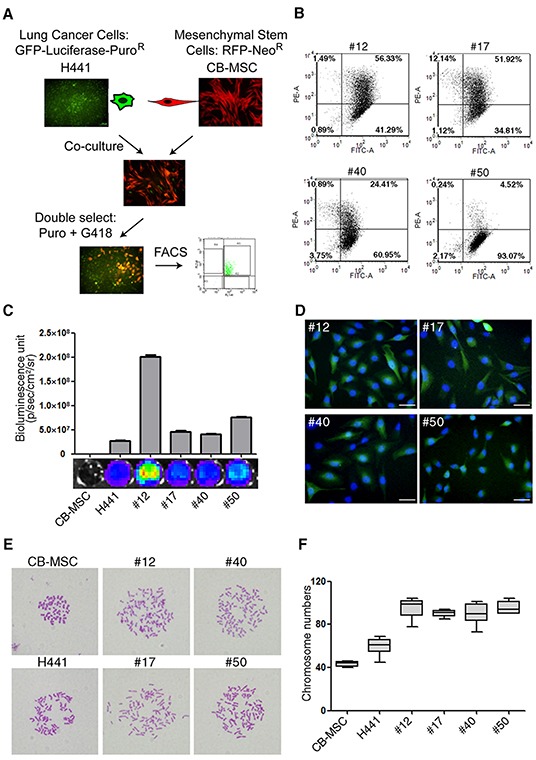
Lung cancer cells fuse spontaneously with MSCs to form synkaryons with full chromosome complements **(A)** Strategy for isolating lung cancer cell-MSC fusion progeny by dual antibiotic selection and dual color FACS. **(B)** Flow cytometric analysis of GFP (FITC-A channel) and RFP (PE-A channel) in fusion progeny; percentages of dual GFP-RFP positive cells are shown in upper right quadrants of each graph. **(C)** Luciferase expression of fusion progeny and parental CB-MSC and H441 cell lines was imaged (below) and quantitated (above) with an IVIS Imaging System. **(D**) Merged fluorescence images of GFP (green) and DAPI (blue) demonstrate that fusion progeny are synkaryons. Scale bars represent 50 μm. **(E)** Representative metaphase spreads for karyotype analysis of fusion progeny and parent cell lines. **(F)** Box-and-whisker plots of chromosome numbers of fusion progeny and parental cell lines calculated by scoring karyotypes. Lines in boxes are medians, and maximum and minimum values are shown by the whiskers.

To generate fusion progeny of MSCs and lung cancer cells we first transduced H441 cells with a green fluorescent protein-firefly luciferase (GFP-Fluc) fusion protein and a puromycin-resistance marker, and CB-MSC cells with red fluorescent protein (RFP) and a neomycin-resistance marker. The product cell lines were then co-cultured without fusogenic agent for 7 days. Spontaneous fusions were selected in medium with puromycin and G418 and further isolated by dual color (GFP and RFP) fluorescence activated cell sorting (FACS) and single cell sub-cloning to generate four fusion cell lines named #12, #17, #40 and #50 (Figure 1A). GFP expression was robust in all fusion lines but RFP and luciferase expression varied within populations of each fusion line (Figure 1B and C). To determine whether variable RFP and luciferase expression resulted from asymmetric chromosome segregation during cell growth in culture, we analyzed DNA content by flow cytometry. We found that DNA content was virtually identical in all fusion cell lines and approximately 2-fold higher than CB-MSC cells (Supplementary Figure S1). The heterogeneous expression of RFP and luciferase may reflect effects of nuclear reprogramming upon fusion. DAPI staining showed that fusion progeny were synkaryons (Figure 1D). Karyotypes of fusion cell lines showed that each was greater than tetraploid, approximating the sum of the H441 and CB-MSC chromosome complements. Not surprisingly, the fusion lines showed greater variability in chromosome number than the diploid CB-MSC cells (Figure 1E and F). Together these results demonstrate that the four progeny cell lines are bona fide fusion products of H441 and CB-MSC cells.

### Growth rate and tumorigenicity of lung cancer cells are reduced upon fusion with MSCs

**Figure 2:**
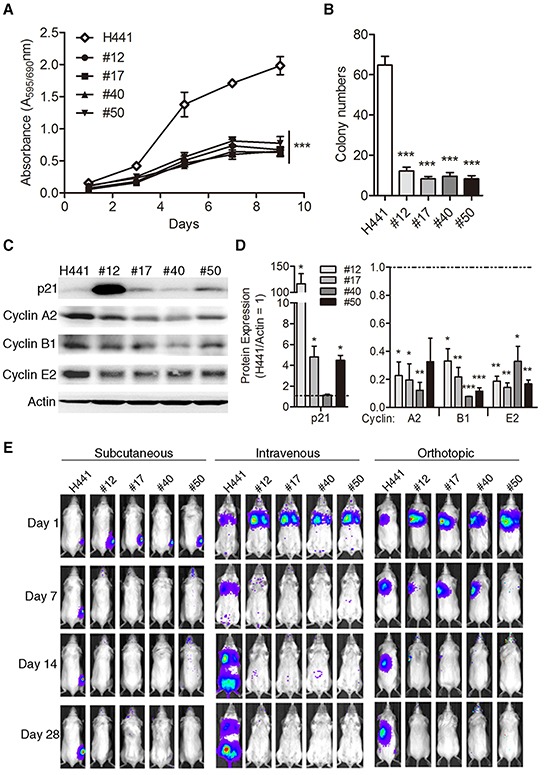
Reduced growth rate, anchorage-independence, and tumorigenicity of lung cancer-MSC fusion progen **(A)** In vitro cell proliferation (mean ± SEM) of fusion progeny and H441 lung cancer cells assessed by MTT assay; *** indicates P<0.001 using two-way ANOVA. **(B)** Anchorage-independent clonogenicity in soft agar. Values are means + SEM; *** indicates P<0.001 in unpaired t-tests with Welch's correction, compared to parental H441 cancer cells. **(C**) Representative western blots showing expression of p21, cyclin A2, cyclin B1, and cyclin E2 in fusion progeny and H441 parent cells, with actin as loading control. **(D)** Quantitative analysis of the relative protein expression of p21, cyclin A2, cyclin B1, and cyclin E2 normalized actin. Values (means + SEM) are normalized to actin loading and are relative to H441 levels (= 1.0; dashed lines). Statistical comparisons of fusion progeny to H441 parent are shown by *, P<0.05; **, P<0.01; and ***, P<0.001 using paired t-tests. **(E)** Representative bioluminescence images from subcutaneous, intravenous, and lung orthotopic xenograft experiments. Images were taken 1, 7, 14, and 28 days after injection of fusion progeny or parental H441 lung cancer cells.

To investigate whether the growth ability of lung cancer cells was influenced by fusion with MSCs, we compared in vitro growth rates of H441 and its MSC fusion progeny by MTT assay. Growth rates of all four fusion progeny were markedly lower than H441 cells (Figure 2A). To evaluate the tumorigenic potential of fusion progeny, we first tested their ability for anchorage-independent growth in soft agar. Parallel to the proliferation results, all four fusion progeny showed 5- to 6-fold reduced clonogenicity in soft agar compared to parental cancer cells (Figure 2B). These changes in growth rate and anchorage-independent growth suggested that fusion altered expression of cell cycle proteins. We explored this possibility by measuring levels of key cell cycle regulatory proteins by western blot. We found that p21 was up-regulated in 3 of 4 fusion progeny, and all four down-regulated cyclins A2, B1, and E2 relative to H441 cells (Figure 2C and D). We next compared the tumorigenic potential of the fusion progeny and parental cancer cells. We inoculated various numbers of cells into severe combined immunodeficiency (SCID) mice by using subcutaneous, intravenous, and orthotopic injections and monitored tumor growth by non-invasive bioluminescent imaging. Representative images are shown in Figure 2E, and quantitative results are shown in Table 1. These data indicate that MSC fusion abolishes the tumorigenic potential of lung cancer cells.

**Table 1:** Quantitation of tumor formation by fusion progeny and parental H441 cancer cells in severe combined immunodeficiency mice

Inoculation model	Cell type	No. Cells (×105)	Tumors/mice
Subcutaneous	H441	2	5/5
	#12	2	0/4
	#12	20	0/6
	#17	2	0/4
	#17	20	0/6
	#40	2	0/4
	#40	20	0/6
	#50	2	0/4
	#50	20	0/6
Intravenous	H441	5	5/5
	#12	5	0/5
	#17	5	0/5
	#40	5	0/5
	#50	5	0/5
Orthotopic	H441	10	5/5
	#12	20	0/5
	#17	20	0/5
	#40	20	0/5
	#50	20	0/5

Animals were implanted with indicated cell numbers in subcutaneous, intravenous, and lung orthotopic models, and the number of mice with tumors after 60 days is indicated.

### Lung cancer-MSC fusion progeny display stem cell-like properties

**Figure 3:**
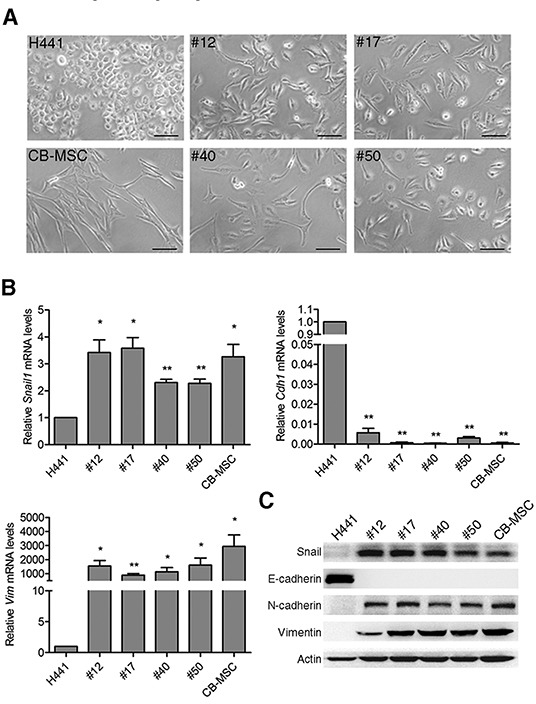
Fusion progeny exhibit epithelial–mesenchymal transition **(A)** Representative phase-contrast microscopic images of fusion progeny and parental cells. Scale bars represent 100 μm. **(B)** mRNA expression of EMT markers Snai1l, Cdh1, and Vim were evaluated by real-time PCR. Values (means + SEM) indicate relative mRNA levels compared to H441 (=1.0) after normalization to eEF1α loading control; * indicates P<0.05 and ** indicates P<0.01 using paired t-tests. **(C)** Western blots of snail, E-cadherin, N-cadherin, vimentin, and actin in fusion progeny and parent cell lines.

Morphological observations showed that parental cancer cells and MSCs display rounded and elongated morphologies, respectively, typical of epithelial and mesenchymal cells, while fusion progeny display the elongated MSC morphology (Figure 3A). The morphological changes reflect that lung cancer-MSC fusion progeny might display traits associated with cells that have undergone an epithelial-mesenchymal transition (EMT). EMT is a type of epithelial plasticity that is characterized by long-lasting morphological and molecular changes in epithelial cells as a result of transdifferentiation towards a mesenchymal cell type. Importantly, cells undergoing an EMT acquire traits of MSCs [39]. Real-time PCR analysis showed fusion progeny up-regulated the EMT transcription factor snail (Snail1) several fold, up-regulated the intermediate filament vimentin (Vim) by ~1000-fold or more, and down-regulated E-cadherin (Cdh1) by >100-fold (Figure 3B). Analysis of protein levels showed corresponding results that fusion progeny display characteristic of MSCs, including up-regulation of snail, N-cadherin, and vimentin and down-regulation of E-cadherin (Figure 3C).

**Figure 4:**
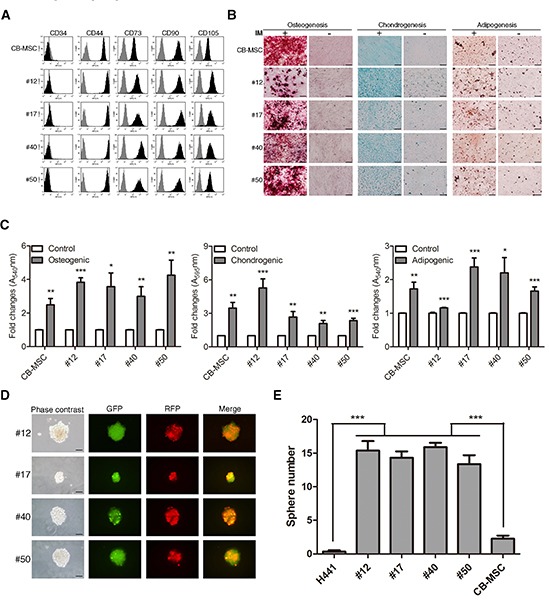
Fusion progeny display stem cell properties similar to MSCs **(A)** Cell-surface marker profiles of fusion and parent MSC cells determined by flow cytometry using antibodies against indicated antigens; grey regions represent isotype controls. **(B)** Multilineage differentiation capacity of fusion progeny and parental MSCs. Osteogenic differentiation was assessed by Alizarin Red S staining for mineral nodule deposition. Chondrogenic differentiation was assessed by Alcian blue staining for proteoglycan deposition. Adipogenic differentiation was assessed by Oil Red O staining for lipid vesicle formation. IM: induction medium. **(C**) Quantitation of multilineage differentiation of fusion progeny and parental MSCs from three independent experiments. Values are means + SEM; *, P<0.05; **, P<0.01; ***, P<0.001 in unpaired t-tests with Welch's correction. **(D)** Representative phase-contrast and fluorescence images and **(E)** quantitation of spheres formed by fusion progeny; scale bars indicate 200 μm. Values are means + SEM; *** indicates P<0.001 in unpaired t test with Welch's correction, compared to H441 or CB-MSC parent cells.

We next determined whether the fusion progeny conserved MSC properties including cell surface markers and differentiation capacity. As shown in Figure 4A, the fusion progeny had similar cell surface marker profiles as MSCs: they were negative for CD34 and positive for CD44, CD73, CD90 and CD105. A key characteristic of MSCs is multipotency. We therefore tested whether the lung cancer-MSC fusion progeny displayed MSC-like multipotency including osteogenic, chondrogenic, and adipogenic differentiation. The fusion progeny and parental MSCs were cultured under standard induction conditions, and in vitro differentiation to distinct mesenchymal lineages was monitored by specific staining. All four fusion progeny showed significant osteogenic and chondrogenic differentiation, and 3 of 4 showed marked adipogenic differentiation comparable to MSCs (Figure 4B and C). MSCs and various tissue-derived stem/progenitor cells form spheres in suspension culture, and sphere formation assay has been extensively utilized to retrospectively recognize stem cells based on their reported capacity to evaluate self-renewal and differentiation at the single-cell level [40]. Representative images of spheres formed by each of the four fusion progeny are shown in Figure 4d. Interestingly, the all four fusion progeny formed spheres more efficiently than the parental H441 cancer cells and MSCs (Figure 4E). These results indicate that fusion progeny lose epithelial traits and maintain most stem-like traits of MSCs, including EMT markers, cell surface markers, significant multipotency, and sphere-forming capacity. Importantly, lung cancer cells are reprogrammed to terminally transdifferentiate into three different lineages of connective tissue cells following fusion with MSC.

### Lung cancer cell transcriptome is reprogrammed upon fusion with MSCs

**Figure 5:**
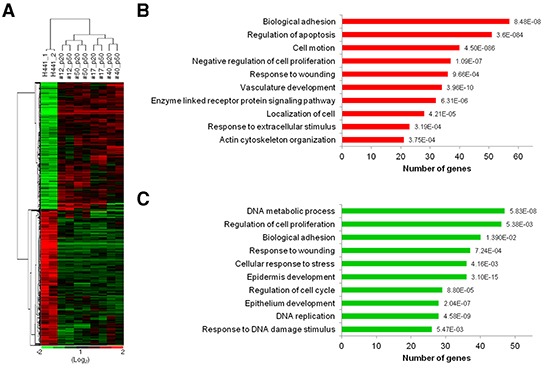
Transcriptional profiling and gene ontology functional analysis of fusion progeny **(A)** Heatmap of hierarchical clustering of 1,475 genes differentially expressed (fold change >1.5) in fusion progeny compared to parental cancer cell (green, down-regulated; red, up-regulated). Transcription profiles were from two independent cultures of H441 cells, and passages 20 and 50 of fusion progeny. Functional annotations of up-regulated **(B)** or down-regulated **(C)** genes in fusion progeny compared to H441 cells. Genes were classified into Gene Ontology biological process categories using DAVID bioinformatics resources. P values for gene-enrichment were calculated using a modified Fisher exact (EASE) score and listed behind each column.

Gene expression patterns from microarray analysis provide a comprehensive view of cellular characteristics. To investigate the difference of transcriptomes between fusion progeny and parental fusion partners, transcription profiles comprising 47,231 genes were determined with Illumina® HumanHT-12 v4 Expression BeadChips. A total of 26,087 genes were expressed at a detectable level (p-value < 0.05) in fusion progeny and parental cells and were subjected to hierarchical clustering. Interestingly, the dendrogram of sample clustering showed that fusion progeny are more closely related to the parental cancer cells than MSCs. Furthermore, transcriptional patterns in fusion progeny from two different passages were very similar, indicating that these cells are quite stable during *in vitro* culture (Supplementary Figure S2). The fact that fusion progeny display many stem-like traits of MSCs but largely retain the transcription profiles of lung cancer cells, suggests that reprogramming toward stemness reflects the effects of relatively a few genes. To further define how lung cancer cells are reprogrammed when fused with MSCs, we focused on 1,475 genes that were differentially expressed (>1.5 fold) in the four fusion progeny relative to the H441 cells, including 722 and 753 that were up- or down-regulated, respectively (Figure 5A). DAVID bioinformatics was used to assign genes into Gene Ontology groups, revealing several important patterns. Consistent with their reduced cell growth, fusion progeny up-regulated apoptosis-related pathway and genes that slow cell proliferation (Figure 5B) as well as down-regulated pathways related to DNA metabolism and replication, cell proliferation, and cell cycle (Figure 5C). Fusion progeny also showed reduced epidermis and epithelium development pathways, which correspond to their EMT features. EMT has been proved to increase cell motility and we did find that fusion progeny up-regulate cell motion and migration (localization) and actin cytoskeleton pathways (Figure 5B). This analysis also suggested fusion progeny were more sensitive to extrinsic stimulation (up-regulating genes that regulate responses to extracellular stimuli and enzyme linked receptor protein signaling pathways) and less resistant to cellular injury (down-regulating DNA damage/stress response pathways) (Figure 5B and C). Collectively, these transcriptional patterns are consistent with the fusion progeny phenotype and support the idea that MSC fusion reprograms lung cancer cells to a more benign state instead of enhanced malignancy.

### FOXF1 facilities reprogramming of lung cancer cells upon MSC fusion

**Figure 6:**
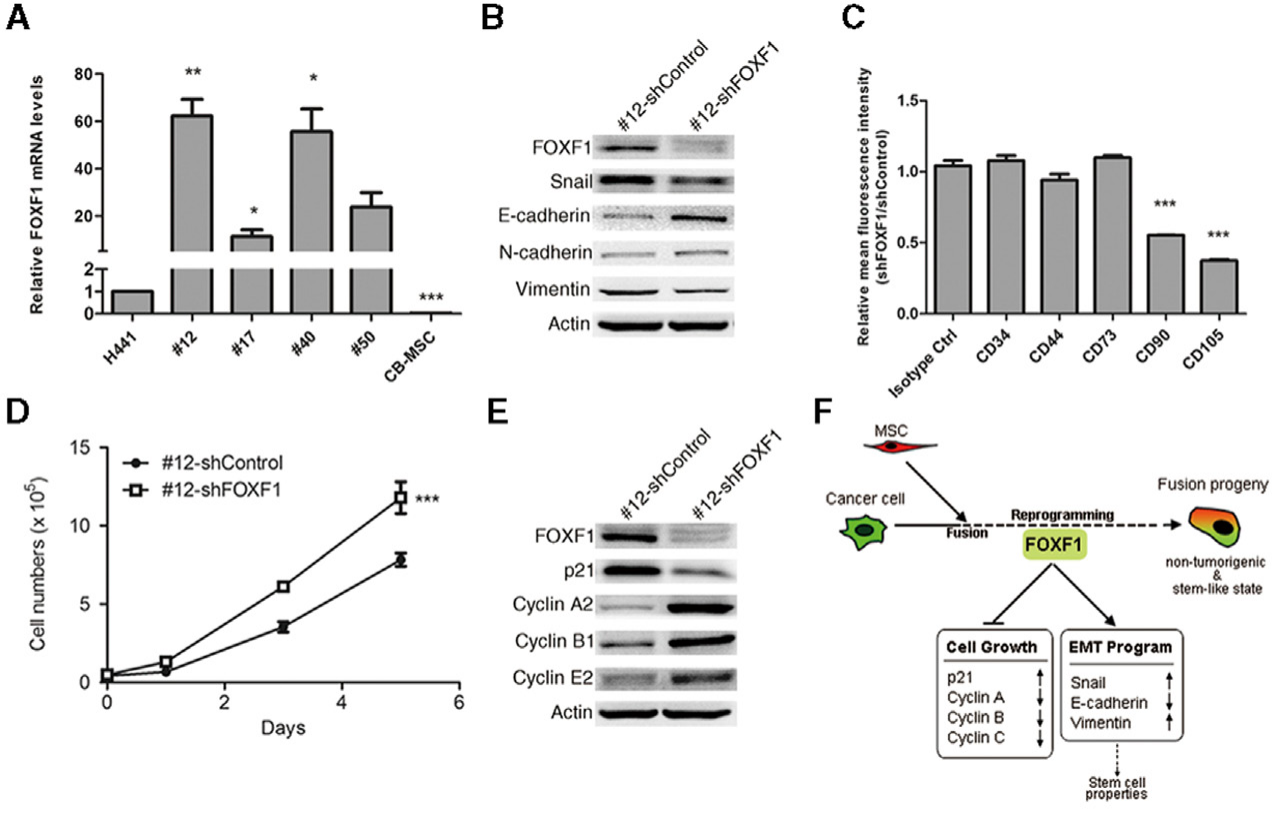
FOXF1 mediates reprogramming effects on fusion progeny **(A)** FOXF1 mRNA levels were determined by real-time PCR in fusion progeny and parent cell lines. Values (means + SEM) are normalized to eEF1α and the H441 level = 1.0; *, P<0.05; **, P<0.01; ***, P<0.001 using paired t-tests. **(B)** Western blots of and EMT proteins in fusion progeny #12 transfected with shFOXF1 or shControl; actin served as loading control. **(C)** Knockdown of FOXF1 reduces MSC-specific cell surface marker expression in fusion progeny #12. Average (+ SEM) fold changes of indicated surface markers in fusion progeny #12 transfected with shFOXF1 and shControl; *** indicates P<0.001 in unpaired t-tests with Welch's correction, compared to isotype control. **(D)** Down-regulation of FOXF1 in fusion progeny #12 increases cell growth. Values are means ± SEM; *** indicates P<0.001 using two-way ANOVA. **(E)** Western blots of cell cycle regulatory proteins in fusion progeny #12 transfected with shFOXF1 or shControl; actin served as loading control. **(F)** A schematic showing that FOXF1 mediates MSC fusion-induced reprogramming of lung cancer cells via regulating cell cycle- and EMT-pathways leading to non-tumorigenic, stem-like state of fusion progeny.

To identify key mediators of transcriptional reprogramming during cell fusion, we identified genes that showed consistent differential expression in fusion progeny vs parental cells (Supplementary Table S1), focusing on transcription factors. Among these factors, the forkhead box F1 (FOXF1) transcription factor was dramatically up-regulated in fusion progeny. Real-time PCR analysis showed that FOXF1 was up-regulated by >10-fold in each fusion cell line, and in subsequent experiments we focused on fusion cell line #12 as it showed the most dramatic changes in FOXF1 expression (Figure 6A). FOXF1 is likely important expressed in mesenchymal cells during embryonic development and plays a critical role in mesenchymal/epithelial induction in various organs [41–42]. To investigate whether FOXF1 plays a key role in reprogramming upon cell fusion, we stably reduced FOXF1 expression using short hairpin RNA (shRNA) and measured expression of key EMT regulatory proteins. FOXF1 knockdown increased the expression of the epithelial marker E-cadherin, and reduced expression of mesenchymal markers snail and vimentin, but not N-cahedrin (Figure 6B), supporting the idea that FOXF1 promotes EMT in fusion progeny. Because EMT is linked to expression of stem cell markers [39], we used FACS to determine whether FOXF1 regulates expression of the stem cell phenotype in fusion progeny #12. We found that FOXF1 knockdown significantly decreased expression levels of MSC markers CD90 and CD105 (Figure 6C). Surprisingly, we found that FOXF1 knockdown significantly enhanced growth rate (Figure 6D), reduced p21 expression, and increased cyclin A2, B1, and E2 expression (Figure 6E). These results implicate FOXF1 in growth suppression of cancer cells after fusion with MSCs. Collectively, FOXF1 plays a regulatory role in mediating the reprogramming effects of MSC fusion on lung cancer cells.

## DISCUSSION

It is well documented that cell fusion is an extremely specialized consequence that occurs in limited condition during development and sexual reproduction [1]. Cell fusion of malignant cells with stromal cells within the tumor microenvironment has been demonstrated in animal models and human cancers [7–8, 11–12, 37–38]. MSC is one of the critical components in the tumor microenvironment and a putative fusion candidate, but the underlying functions of MSC fusion with malignant cells remain poorly understood. Validation of cell fusion has proven difficult, leading to the development of dual selection/screening strategies [33–34, 43]. Using a dual antibiotic/dual fluorescence marker strategy we found that lung cancer cells and MSCs fused spontaneously, that fusion progeny contained essentially complete chromosome complements of parental cells, and consistent with previous reports, they exhibited markedly different phenotypes from parental cells [43–44]. We observed a degree of variation among fusion products (i.e., variable expression of RFP) that may reflect variation in nuclear reprogramming via genetic or epigenetic effects. Nonetheless, independent fusion progeny shared critical characteristics such as reduced growth rate, and altered expression of genes that regulate cell cycle, EMT, and stem cell properties. These changes appear to reflect MSC-driven reprogramming of lung cancer cells since the fusion transcriptomes were more similar to lung cancer cells than to MSCs (Supplementary Figure S2).

In early studies fusion between normal fibroblasts and malignant cells suppressed tumorigenicity through cell cycle effects [45], and recent studies of stem/cancer fusions led to similar conclusions [36, 46], but the precise underlying mechanisms remain elusive. The cyclin-dependent kinase inhibitor p21 is a p53-downstream target and a key mediator that promotes p53-dependent cell cycle arrest in response to many stimuli [47]. However, subsequent studies showed that p21 plays a crucial role in multiple tumor suppressor pathways for promoting anti-proliferative activities that are independent of the classical p53 tumor-suppressor pathway [48]. Hence, deregulated p21 has been related to many pathological symptoms such as carcinogenesis, senescence, and age-related diseases. The H441 lung cancer cell line expressed low levels of p21 and MSC fusion increased p21 expression as well as reduced cell growth. We propose that the anti-malignant effects of MSC fusion on lung cancer cells reflect the reprogramming potential of MSCs via complementation of tumorigenic defect such as deregulated p21 herein. Although p21 is not a transcription factor, it has been proved that its biological functions are mediated by regulating cellular gene expression [49]. To demonstrate the p21 function were restored in fusion progeny, we screened the expression of p21-regulated genes, which were previously identified by Chang et al [49], in microarray analysis. From a list of 122 p21-regulated genes we found 112 genes in our microarray analysis were expressed at a detectable level in fusion progeny and parental cells (Supplementary Table S2 and Figure S3). Fifty-eight of 69 (84%) genes were down-regulated and 29 of 43 (67%) genes were up-regulated in the four fusion cell lines relative to the H441 cell. Most of the down-regulated genes expressed differentially in fusion progeny were associated with mitosis, cell-cycle progression, and DNA repair. In addition, several up-regulated genes were involved in the regulation of apoptosis and senescence. These differentially expressed genes indicated that the anti-malignant effects of MSC fusion on lung cancer cells may be accomplished by restoring the p21 expression. In addition to the anti-malignant effects, microarray analysis indicated that fusion progeny exhibited more benign characteristics including increased sensitivity to extrinsic stimulation, and reduced resistance to cellular injury. Taken together, these transcriptional patterns are consistent with the fusion progeny phenotypes and support the idea that MSC fusion reprograms lung cancer cells to a more benign state via restoration of p21 function.

Cellular reprogramming is the functional conversion of differentiated somatic cells to pluripotent or multipotent states. Stem cell fusion can reset and regulate the phenotypic characteristics and differentiated state of somatic cells and then reprogram them to stem- or progenitor-like cells [30–31]. MSCs contribute to nonmesodermal tissues such as heart, lung, liver, and intestine [50–51], reflecting stem cell plasticity termed transdifferentiation ability, which has been linked to somatic cell reprogramming via stem cell fusion [31], including transdifferentiation ability of adult stem cells [28–29]. It is, however, unclear whether malignant cells can be similarly reprogrammed by MSC fusion. In our study, the fusion progeny displayed MSC characteristics, including EMT markers, specific surface markers, and sphere-forming capacity. Importantly, we demonstrated that lung cancer cells are reprogrammed with multipotent differentiation ability upon MSC fusion and can be subsequently terminally transdifferentiated into three different lineages of connective tissue cells. A recent study has proved that reprogrammed sarcoma with pluripotent transcription factors lost their tumorigenicity and dedifferentiated to stem-like cells that can be terminally differentiated into mature cell types [52]. The reprogramming may overcome the multitude of genetic aberrancies inherent in malignant cells to restore normal terminal differentiation pathways. It suggests that malignant cells can be reversed from their current tumorigenicity stage to a normal or terminal differentiation stage.

The dramatic differences in the transcriptomes of fusion progeny compared to parent cells implicated transcription factors in mediating these changes. We focused on FOXF1 because it was consistently up-regulated >10-fold in fusion progeny and it has important roles in tissue development. FOXF1 is involved in mesenchyme development of several organs [41–42], homeostasis and repair of lung tissue [53], and it is a unique marker of tissue-specific mesenchymal progenitor cells in human lung [54]. We found that FOXF1 has a critical role in regulating fusion progeny phenotypes, as FOXF1 knockdown reversed key MSC characteristics in fusion progeny including EMT programs and specific surface markers. Furthermore, we also revealed that FOXF1 significantly reduced growth rate and the expression levels of proteins that regulate cell cycle. Hence, in addition to the reprogramming toward stemness, FOXF1 contributes to the anti-malignant effects of MSC fusion on lung cancer cells by regulating the expression of p21. FOXF1 was first implicated as a tumor suppressor when it was shown to be expressed at low levels in prostate cancer [55]. It is also epigenetically silenced in breast cancer, and a regulator of cell cycle progression and the p53-p21 checkpoint pathway that maintains genome stability [56–57]. Moreover, *in silico* analysis of FOXF1 expression showed that FOXF1 is significantly underexpressed in malignant lung tissues compared to normal lung tissues (by at least 3.6 fold) (Supplementary Figure S4). These results implicate that FOXF1 may behave as a tumor suppressor gene in lung cancer.

In conclusion, we demonstrated that MSCs fuse spontaneously with lung cancer cells causing reprogramming to a slow-growing, non-tumorigenic, stem-like state. The anti-malignant effects of MSC fusion-induced reprogramming on lung cancer cells were accomplished by complementation of genetic defects, including up-regulation of FOXF1 and p21 as well as restoration of normal terminal differentiation pathways. FOXF1 behaved not only as a reprogramming regulator that mediates stemness but also as a putative tumor suppressor that contributes to p21-regulated growth suppression during fusion process (Figure 6F), suggesting that FOXF1 and its downstream effectors may be valuable as molecular targets for the further development of diagnostic and therapeutic tools in lung cancer. Much work yet needs to be done to fully understand the consequences of MSC fusion with malignant cell. However, we propose the concept that malignant cells can be reversed from an aggressive state to a more benign or normal-like state upon fusion with MSCs. Furthermore, the fact that MSC fusion reverses phenotypes of malignant cells raises the possibility that in addition to targeting FOXF1 pathways, therapeutic benefits may be realized by targeting factors that regulate cell fusion.

## MATERIALS AND METHODS

### Cell lines and cell fusion

H441 (ATCC-HTB 174) lung cancer cells were infected with SFG-GL and pBABE-puro (Addgene) retroviral particles and cultured in RPMI 1640 medium supplemented with 10% fetal bovine serum, 100 units/mL penicillin, 100 μg/mL streptomycin, 0.25 μg/mL amphotericin B, 1% MEM non-essential amino acids solution, and 2 μg/mL puromycin in a humidified atmosphere with 5% CO_2_ at 37°C to stably express eGFP and Fluc. CB-MSC cells were provided by Dr. Shiaw-Min Hwang from Bioresource Collection and Research Center, Food Industry Research and Development Institute, Hsinchu, Taiwan. CB-MSC cells were collected from umbilical cord blood and transfected with hTERT expression vector pGRN145 and pDsRed-N1 for immortalization and RFP expression [58]. The cells were cultured in αMEM supplemented with 10% fetal bovine serum, 100 units/mL penicillin, 100 μg/mL streptomycin, 0.25 μg/mL amphotericin B, 4 ng/mL basic fibroblast growth factor, 30 μg/mL hygromycin B, and 200 μg/mL geneticin in a humidified atmosphere with 5% CO_2_ at 37°C. To obtain fusion progeny, we co-cultured 2 × 10^5^ CB-MSCs with 10^5^ H441 cells for 7 days without any fusogenic agent. We then selected the spontaneous fusion products with medium containing puromycin and G418 and typically obtained 20-40 fusion clones after dual antibiotic selection. The fusion progeny were further isolated by dual color (GFP and RFP) using FACS and single cell sub-cloning.

### Animal studies

All animal studies were approved by the Institutional Animal Care and Use Committee of Taipei Medical University. Four- to six-week-old female SCID mice were purchased from National Taiwan University (Taipei, Taiwan). The mice were housed under pathogen-free conditions and fed autoclaved food and water. For tumorigenicity experiments, animals were implanted with H441 cells and four fusion cell lines via subcutaneous, intravenous, and lung orthotopic injection.

### Bioluminescence imaging (BLI)

BLI of cells and animals was performed with an IVIS Imaging System 200 Series (PerkinElmer) and quantitated with Living Image® software by measuring photon flux (photons/s/cm2/steradian) in regions of interest drawn around appropriate signals. For *in vitro* BLI, 10^5^ H441, CB-MSC or fusion progeny cells were resuspended in 50 μL PBS and placed in 96-well black imaging plates, and 50 μL D-Luciferin reagent (1.5 mg/mL) (Gold Biotechnology) were added to each well and mixed well, and 30 sec later BLI was performed and the signal was acquired for 1 min. For *in vivo* BLI, anesthetized mice were injected intraperitoneally with 75 mg/kg of D-Luciferin and images were acquired 2–5 min after injection. Acquisition times were 2 min initially and were reduced in accordance with signal intensity to avoid saturation.

### Karyotype analysis

Cells were treated with 0.05 μg/mL demecolcine (Sigma-Aldrich) in growth medium for 1 h at 37°C, 5% CO_2_, harvested to single-cell suspensions with trypsin, washed in PBS, pelleted by low speed centrifugation, resuspended in 0.7% (w/v) sodium citrate, and incubated at 37°C for 25 min. The cells were then fixed in 3:1 methanol/acetic acid and dropped on slides to create chromosome spreads. Slides were stained with 5% Giemsa stain solution (Gibco) for 20 min, washed twice with distilled water, mounted, and evaluated with light microscopy.

### In vitro cell growth rate assay

Cell viability was determined with MTT assay using Thiazolyl Blue Tetrazolium Bromide (Sigma-Aldrich). Cells (1 × 10^3^) were seeded into 96-well plates using 8 wells/cell line/time point. The MTT reagent was added into each well on days 1, 3, 5, 7, and 9. O.D. values (O.D. 595~O.D. 690) were analyzed 4 h after addition of MTT reagent by using a Multiskan PC (Thermo Labsystem). For cell counting assay, 5 × 10^4^ cells were seeded into each well in 6-well plates using 3 wells/cell line/time point, and data were collected on day 0, 1, 3, and 5.

### Anchorage-independent growth

Base agar comprised 1 mL of 0.6% agar in complete growth medium in each well of 6-well plates. Soft agar comprising 1 mL of 0.35% agar in complete growth medium and 1 × 10^4^ cells was overlaid on base agar. Cells were incubated at 37°C for 2 weeks, and resulting colonies were counted after staining with 10% crystal violet in methanol (Fisher Scientific).

### Flow cytometry

To purify GFP+/RFP+populations of fusion progeny, cells with dual resistance to G418 and puromycin were sorted using a FACSAria III flow cytometer (BD Biosciences). Briefly, cells were first sorted at a high rate (10,000-20,000 cells/sec) using a GFP+/RFP+ gate that captured approximately 10% of viable cells; these were then resorted at a slower rate (1-200 cells/sec) to obtain highly purified populations.

For DNA content analysis, cells were harvested, resuspended in Hank's balanced salt solution (HBSS; Gibco®) (1 × 10^6^ cells/mL), and 1 mL aliquots of cell suspensions were incubated with 5 μM DyeCycle™ Violet (Molecular Probes) at 37°C for 30 min and protected from light. After incubation, cells were washed and analyzed using ~405 nm excitation and ~440 nm emission.

For cell surface marker analysis, cells were harvested, resuspended in 100 μL HBSS containing specific antibodies, and incubated at 4°C for 30 min. Antibodies were used at concentrations as recommended by the manufacturer. Cell surface antibodies were conjugated with allophycocyanin (APC) and purchased from eBioscience, including mouse monoclonal anti-human CD34 (#170349), rat monoclonal anti-human CD44 (#170441), mouse monoclonal anti-human CD74 (#170739), mouse monoclonal anti-human CD90 (#170909), and mouse monoclonal anti-human CD105 (#171057). Mouse IgG (#174714) and rat IgG (#174321) were included as isotype controls.

GFP, RFP, DNA content, and surface markers were analyzed using FACSCanto II low cytometer (BD Biosciences) and FCS Express software (De Novo).

### Real-time PCR

Total RNA was extracted from cells using High Pure RNA Isolation Kit (Roche) according to the manufacturer's instructions. Reverse transcription (RT) was performed as previously described [59]. Quantitative real-time PCR was performed using an ABI 7300 real-time PCR system (Applied Biosystems), and gene expression was calculated by the 2^−ΔCt^ or 2^−ΔΔCt^ methods with calibration samples included in each experiment.

The primers used were:

Snai1l -F: 5′-TCGGAAGCCTAACTACAGCGA-3′

Snai1l -R: 5′-AGATGAGCATTGGCAGCGAG-3′

Cdh1-F: 5′-CGCCCTATGATTCTCTGCTCG-3′

Cdh1-R: 5′-TCGTCCTCGCCGCCTCCGTA-3′

Vim-F: 5′-CCATCAACACCGAGTTCAAGAA-3′

Vim-R: 5′-GGCCAAGCGGTCATTCAG-3′

FOXF1-F: 5′-AAGCCGCCCTATTCCTACATC-3′

FOXF1-R: 5′-GCGCTTGGTGGGTGAACT-3′

eEF1α-F: 5′-CACACGGCTCACATTGCAT-3′

eEF1α-R: 5′-CACGAACAGCAAAGCGACC-3′

### Western blot analysis

The protein extraction and immunoblotting were performed as previously described [60]. The following antibodies were used: rabbit polyclonal anti-FOXF1 (Abcam #ab23194, 1:500), rabbit monoclonal anti-p21 (Cell Signaling Technology #2947, 1:2000), mouse monoclonal anti-Cyclin A2 (Cell Signaling Technology #4546, 1:1000), rabbit polyclonal anti-Cyclin B1 (Cell Signaling Technology #4138, 1:1000), rabbit polyclonal anti-Cyclin E2 (Cell Signaling Technology #4132, 1:750), rabbit monoclonal anti-Snail (Cell Signaling Technology #3879, 1:500), rabbit monoclonal anti-E-cadherin (GeneTex #GTX61329, 1:4000), rabbit monoclonal anti-N-cadherin (Epitomics #2447, 1:500), rabbit polyclonal anti-Vimentin (GeneTex #GTX100619, 1:4000), and mouse monoclonal anti- Actin (Millipore #MAB1501, 1:10000).

### Multilineage differentiation Assays

To evaluate the *in vitro* differentiation potential of cells, we conducted differentiation induction experiments of three major mesodermal lineages. Briefly, cells were seeded in 6-cm tissue culture dishes to 80-90% confluence. For osteogenic differentiation, cells were cultured in α-MEM supplemented with 10% FBS, 0.1 μM dexamethasone, 10 mM β-glycerophosphate, and 50 mM ascorbic acid for 21 days, and cells were stained with 2% Alizarin Red S (pH 4.2) for 15 min at room temperature. Dishes were photographed then bound stain was eluted with 10% cetylpyridinium chloride and quantified by spectrophotometric absorbance at 540 nm. For chondrogenic differentiation, cells were cultured in α-MEM supplemented 10% FBS, 10 ng/mL TGF-b1, 10 nM dexamethasone for 21 days, and stained with 1% Alcian blue 8GX reagent in 3% glacial acetic acid (pH 2.5) for 30 min at room temperature. Dishes were photographed, and then bound stain was eluted with dissociation solution (4M guanidine-hydrochloride and 33% propanol) and quantified by spectrophotometric absorbance at 595 nm. For adipogenic differentiation, cells were cultured in α-MEM supplemented with 10% FBS, 1 μM dexamethasone, 10 μg/mL insulin, and 0.5 mM 3-methyl-1-isobutylxanthine for 2 days, then cells were incubated for 21 days in maintenance medium (α-MEM, 10% fetal calf serum, and 10 μg/mL insulin). Cells were fixed and stained with 0.5% oil red O in 60% isopropyl alcohol for 15 min to detect lipid droplets. Dishes were photographed, and then lipid droplets were extracted with 100% isopropyl alcohol and quantified quantified by spectrophotometric absorbance at 540 nm.

### Sphere Formation Assay

H441, CB-MSC, and fusion progeny cells were incubated for 14 days in 1 mL of modified sphere medium (DMEM/F12 medium supplemented with 1X B-27 supplement (Gibco), 20 ng/mL epidermal growth factor (PeproTech), 10 ng/mL fibroblast growth factor-basic (PeproTech), and 20 ng/mL human leukemia inhibitory factor (Sigma-Aldrich) in 6-well plates (10^4^ cells/well). Spheres (>100 μm diameter) were counted and photographed.

### Microarray analysis

Total RNA was prepared with the High Pure RNA Isolation Kit (Roche), and transcription profiles were generated using HumanHT-12 v4 Expression BeadChip (Illumina) by Health GeneTech Corp. services. The quality of each RNA sample was assessed using an Aglilent 2100 Bioanalyzer and NanoDrop-1000. RNA was amplified and labeled using the Ambion Illumina TotalPrep RNA Amplification kit (Invitrogen) and the TotalPrep RNA Labeling Kit (Ambion). The samples were hybridized for 16 h at 58°C to a HumanHT-12 v4 Expression BeadChip. After hybridization, the chip was washed, stained, and scanned with an iScan scanner. Microarray data were analyzed using GenomeStudio software version 2011.1 (Illumina) and normalized by Gene Expression Module software version 1.9.0 (Illumina). The hierarchical clustering and heatmap of differentially expressed genes were generated using Cluster 3.0 software and Java TreeView software version 1.1.4r4. After clustering, the functional annotations of transcripts were determined using DAVID Bioinformatics Resources v6.7 [61–62].

### shRNA knockdown of FOXF1

FOXF1 mRNA was knocked down using the GIPZ Lentiviral shRNAmir system. Six shRNAmir constructs (GIPZ Human FOXF1 shRNA clone IDs: V3LHS_392861, V2LHS_131754, V3LHS_413433, V3LHS_413431, V2LHS_131753, and V2LHS_131755) were tested along with a shRNAmir control vector (Open Biosystems). Lentiviral vectors and packaging constructs were transfected into 293FT cells (Invitrogen) with Lipofectamine 2000 Transfection Reagent (Invitrogen). Infectious viral particles were collected 48 h after transfection. Log-phase target cells were infected with appropriate virus titers in media containing 8 μg/mL polybrene. Media was changed the following day, and 24 h later transfectants were selected with puromycin (4 μg/mL) for 5 days, and subsequently cultured in complete growth medium with 2 ug/mL puromycin. Real-time and western blot analyses were utilized to evaluate the degree of FOXF1 silencing. The most effective FOXF1 knockdown was achieved with the V2LHS_131755 GIPZ Human FOXF1 shRNAmir construct.

### Statistical analysis and replicates

The sizes of sample group in all data are at least n = 5, unless otherwise indicated. All data presented are representative of at least three independent experiments that yielded similar results. Statistical analyses were performed using GraphPad Prism 5.

## SUPPLEMENTARY FIGURES AND TABLES


