# Supplementary material for: FOXF1 mediates mesenchymal stem cell fusion-induced reprogramming of lung cancer cells
Source: Oncotarget. 2014 Sep 9;5(19):9514–29. doi: 10.18632/oncotarget.2413 (PMC4253450; doi:10.18632/oncotarget.2413)
Supplement: Supplementary file 1 [file oncotarget-05-9514-s001.pdf]

SUPPLEMENTARY FIGURES AND TABLES

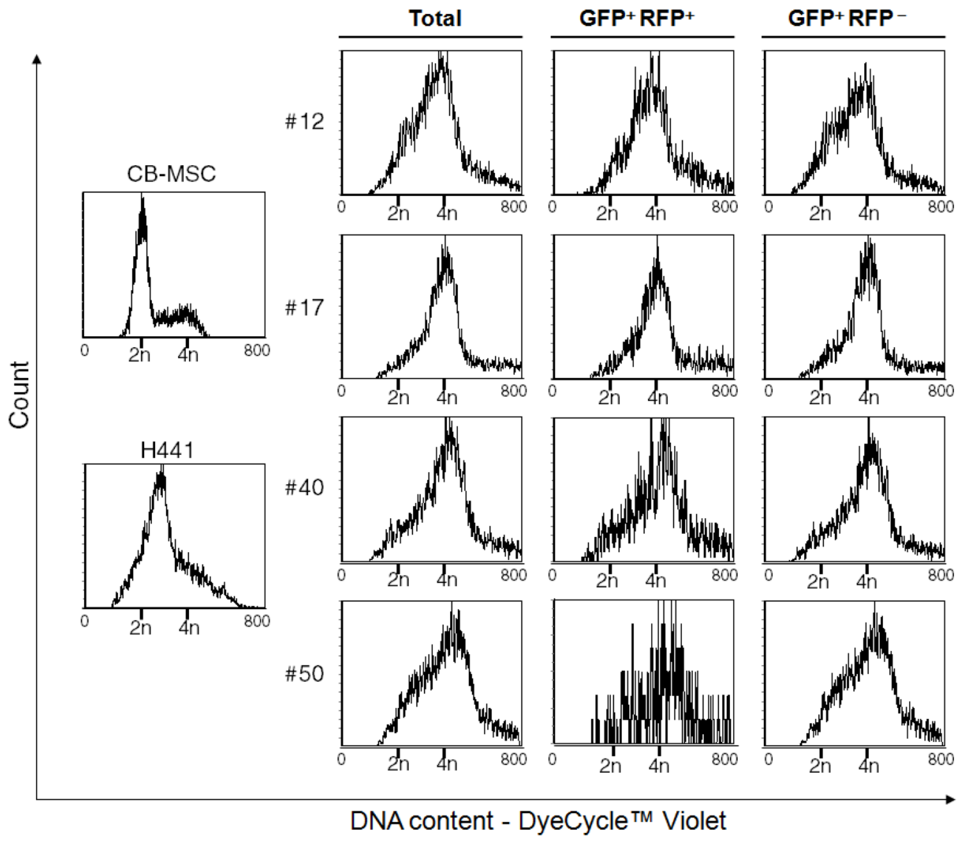

Supplementary Figure S1: DNA content in distinct fluorescent cell populations of fusion progeny. The DNA content of fusion progeny and parental cells were analyzed by DNA staining with Dye-Cycle Violet and flow cytometry. DNA content of GFP+/RFP+ and GFP+/RFP- populations of fusion progeny had nearly identical DNA content, and DNA content of all fusion progeny was >2-fold higher than parental MSCs. Parental MSC are diploid control (n = haploid chromosome number of CB-MSC).

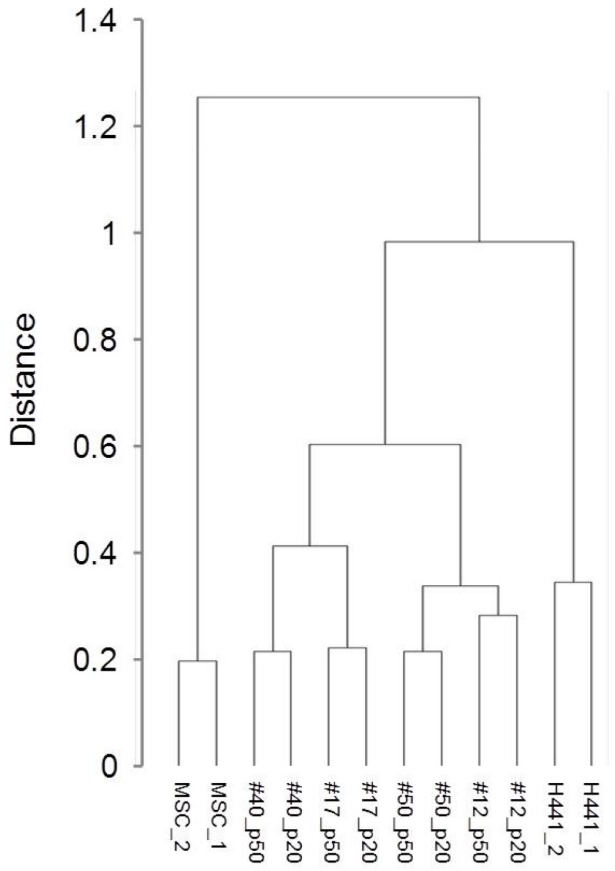

Supplementary Figure S2: Hierarchical clustering dendrogram of the transcriptomes of fusion progeny and parental fusion partners. Based on present/absent signals, we found 26,087 transcripts found in fusion progeny and their parental partners with at least one significant expression (detection p-value < 0.05). RNA was extracted from two independent cultures of each parental cell line (H441_1, H441_2 and MSC_1, MSC_2) and passages 20 and 50 of each fusion progeny. Pearson's correlation distance was used as the metric to create the transcriptional profile dendrogram.

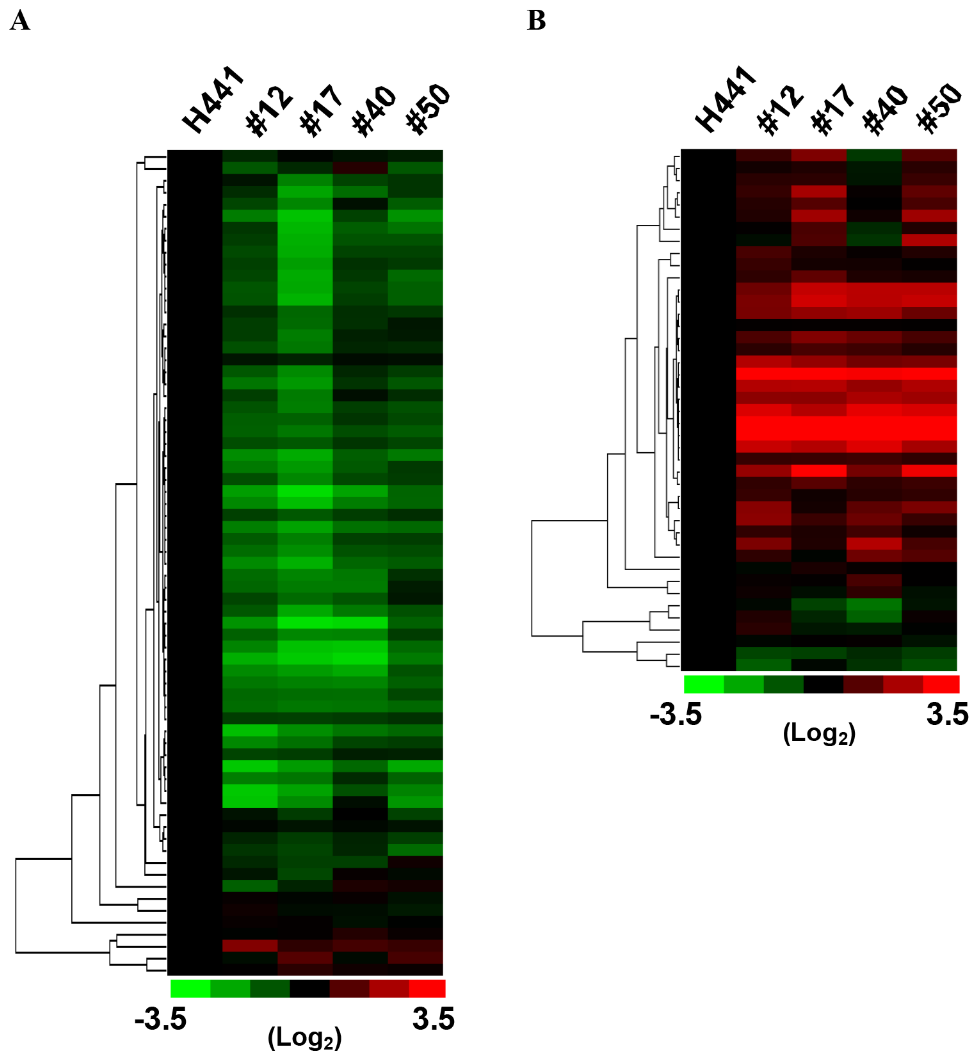

Supplementary Figure S3: Heatmap of p21-regulated genes. From a list of seventy-four p21-inhibited genes and forty-eight p21-induced genes, which were previously identified by Chang et al [1], we found **(A)** sixty-nine p21-inhibited genes and **(B)** forty-three p21-induced genes in our microarray analysis were expressed at a detectable level in fusion progeny and parental cells. Values indicate the log2 fold change of gene expression in four fusion progeny relative to H441 cells.

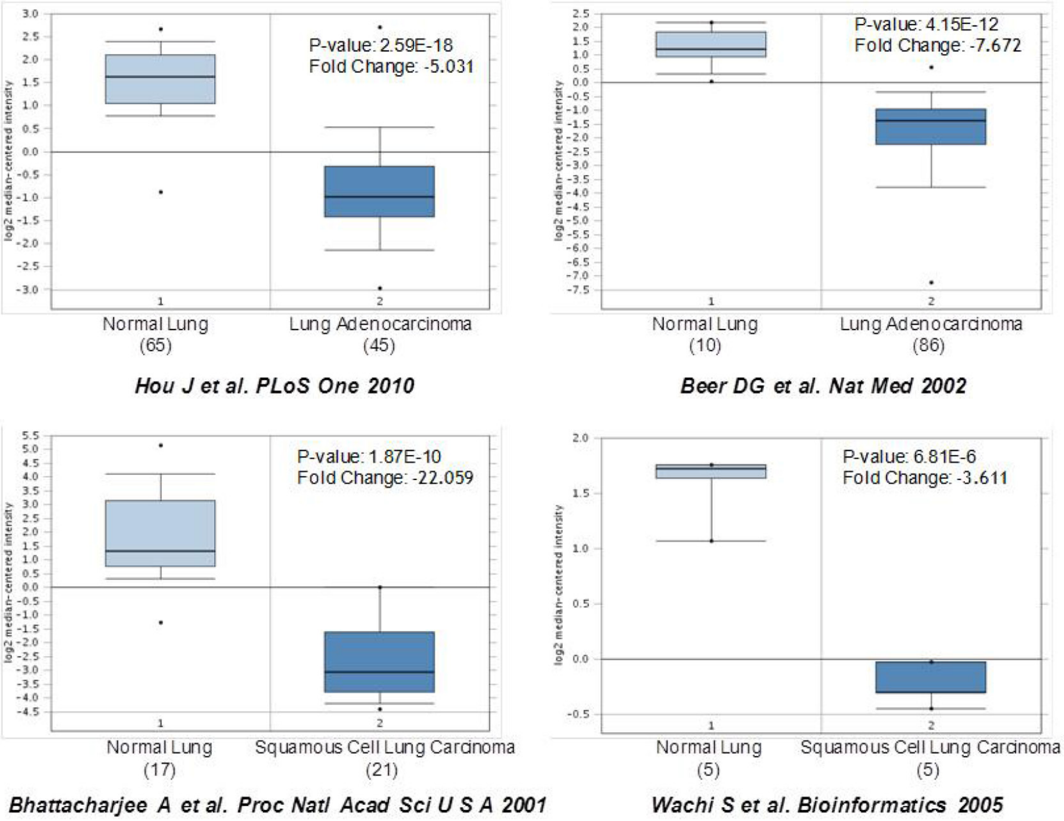

Supplementary Figure S4: *In silico* analysis of *FOXF1* expression. We performed *in silico* analysis of *FOXF1* expression using Oncomine Research Edition (https://www.oncomine.org/). *FOXF1* mRNA expression was queried in cancer vs. normal in the lung cancer category. The query threshold was set at a p-value $<10^{-4}$, over two-fold difference of expression, and top 10% ranking in under-expression genes. We found four analyses from four studies met these criteria.[2–5] *FOXF1* mRNA expression in normal and malignant lung specimens are presented as box and whisker plots. Sample numbers, fold changes, and P values for *FOXF1* expression between normal and malignant specimens are indicated.

Supplementary Table S1. Genes showing consistent differential expression in fusion progeny versus parental cells

TargetID	SOURCE_REFERENCE_ID	ProbeID	Average Signal						Signal Ratio Relative to H441				
			H441	CB-MSC	#12	#17	#40	#50	CB-MSC	#12	#17	#40	#50
CHN1	NM_001025201.1	610332	215.82	211.54	536.36	834.76	750.03	495.26	0.98	2.49	3.87	3.48	2.29
FOXF1	NM_001451.2	3800554	115.20	126.11	1687.40	872.02	1051.02	1086.15	1.09	14.65	7.57	9.12	9.43
HS.537002	Hs.537002	2750202	156.56	162.32	924.71	416.65	734.94	517.27	1.04	5.91	2.66	4.69	3.30
IGFN1	NM_178275.4	840128	121.06	120.38	406.18	328.99	288.31	653.41	0.99	3.36	2.72	2.38	5.40
LOC652683	XM_942283.2	3800692	142.23	138.70	3413.31	3888.32	379.18	2382.76	0.98	24.00	27.34	2.67	16.75
LOC653071	XM_930721.1	6760343	141.08	141.72	635.27	413.68	466.02	719.68	1.00	4.50	2.93	3.30	5.10
NPC1	NM_000271.3	7570358	594.92	615.70	1667.17	1295.71	2159.39	1328.40	1.03	2.80	2.18	3.63	2.23
PLAC8	NM_016619.1	4830113	253.27	247.88	1839.59	1873.64	1261.08	2659.26	0.98	7.26	7.40	4.98	10.50
PSG4	NM_002780.3	3400474	140.00	144.07	1013.37	501.48	2573.65	1076.95	1.03	7.24	3.58	18.38	7.69
PSG9	NM_002784.2	4120243	116.29	122.03	1051.33	454.58	3344.36	910.10	1.05	9.04	3.91	28.76	7.83
SFRP1	NM_003012.3	6960379	147.00	149.80	366.39	573.08	523.34	755.09	1.02	2.49	3.90	3.56	5.14
SPANXA2	NM_145662.2	6960653	160.95	153.91	3231.63	4295.63	408.10	2078.12	0.96	20.08	26.69	2.54	12.91
SPANXB2	NM_145664.1	7330280	128.51	123.64	3229.78	3962.10	324.25	2021.64	0.96	25.13	30.83	2.52	15.73

Thirteen genes were consistently under- or over-expressed in fusion progeny relative to parental cell lines. For these genes, expression ratios of CB-MSC relative to H441 were between 0.95-fold and 1.05-fold. For fusion progeny all ratios were >2.

Supplementary Table S2. Summary of genes regulated by p21

ENTREZ GENE_ID	TargetID	Fold change relative to H441 (log2)			
		#12	#17	#40	#50
p21-inhibited genes					
983	CDC2	-1.04	-2.00	-1.44	-0.95
1163	CKS1B	-2.07	-2.40	-2.55	-1.45
5347	PLK1	-1.14	-1.41	-0.92	-1.08
23397	NCAPH	-0.48	-0.07	-0.22	-0.35
1058	CENPA	-1.11	-1.15	-0.62	-0.85
1063	CENPF	-0.72	-1.26	-0.56	-0.27
4085	MAD2L1	-1.87	-2.61	-1.95	-1.18
701	BUB1B	-0.98	-1.50	-0.74	-0.94
11004	KIF2C	-0.96	-1.41	-0.44	-0.50
3833	KIFC1	-0.87	-2.04	-0.80	-0.79
1653	DDX1	-0.22	-0.71	0.00	-0.78
6790	AURKA	-0.82	-1.59	-0.20	-1.09
9212	AURKB	-2.37	-1.84	-1.23	-2.00
23082	PPRC1	-0.91	-1.05	-0.63	-0.82
11113	CIT	-0.25	-0.42	-0.13	-0.17
4001	LMNB1	-1.43	-1.81	-1.06	-0.67
84823	LMNB2	-0.23	-1.53	-0.80	-0.61
7112	TMPO	-0.84	-2.02	-0.67	-1.24
2305	FOXM1	-0.65	-2.17	-1.10	-1.36
7083	TK1	-0.76	-1.87	-0.58	-0.69
7298	TYMS	-1.04	-1.63	-0.45	-0.71
7378	UPP1	-0.03	0.06	0.42	0.11
6240	RRM1	-0.72	-1.48	-0.39	-0.30
6241	RRM2	-1.47	-2.30	-0.78	-1.74
4176	MCM7	-1.61	-1.32	-0.80	-0.73
4173	MCM4	-1.61	-2.25	-1.49	-1.20
8318	CDC45L	-1.75	-2.65	-2.55	-1.15
4998	ORC1L	-1.29	-1.68	-0.97	-0.90
5422	POLA1	-0.93	-1.60	-0.86	-0.72
5984	RFC4	-1.27	-1.57	-1.43	-0.58
4605	MYBL2	0.09	-0.06	0.10	-0.20
9319	TRIP13	-1.01	-1.99	-0.81	-1.13
7153	TOP2A	-0.72	-2.09	-0.99	-0.99
8208	CHAF1B	-0.48	-0.75	-0.75	0.19

ENTREZ GENE_ID	TargetID	Fold change relative to H441 (log2)			
		#12	#17	#40	#50
3146	HMGB1	-1.29	-1.41	-1.38	-1.24
3148	HMGB2	-1.21	-1.45	-1.46	-0.33
2189	FANCG	-2.25	-1.68	-1.39	-1.22
8438	RAD54L	-0.82	-1.26	-1.01	-0.28
9156	EXO1	-1.64	-1.84	-2.01	-0.97
3978	LIG1	-0.53	-1.95	-1.28	-0.62
5885	RAD21	-0.99	-2.11	-0.73	-1.13
10915	TCERG1	-0.56	-1.22	-0.55	-0.66
10189	THOC4	-1.50	-1.93	-1.34	-1.23
10322	SMYD5	-0.08	-0.24	-0.10	-0.22
2146	EZH2	-1.07	-1.50	-0.76	-0.73
6421	SFPQ	0.18	-0.14	-0.15	-0.30
3184	HNRNPD	-0.76	-1.05	-0.72	-0.54
10465	PPIH	-1.42	-1.53	-1.58	-1.12
26227	PHGDH	-1.13	-0.41	0.35	0.24
8140	SLC7A5	-2.32	-1.65	-0.16	-1.77
3161	HMMR	-0.25	-0.84	0.09	-0.11
9787	DLG7	-0.74	-1.46	-0.18	-0.53
10293	TRAIP	-0.73	-0.76	-0.70	-0.58
10797	MTHFD2	-1.24	-1.30	-1.30	-0.84
6520	SLC3A2	-1.03	-0.48	0.44	-1.02
9656	MDC1	-1.50	-1.38	-0.49	-1.17
27346	TMEM97	-1.15	-1.60	-1.56	-0.72
5612	PRKRIR	0.10	0.06	-0.18	-0.03
2542	SLC37A4	-0.14	0.99	-0.12	0.83
27101	CACYBP	-2.37	-1.93	-0.95	-1.55
4953	ODC1	-0.62	-0.81	-0.45	-1.26
10024	TROAP	-1.38	-1.80	-0.61	-1.02
6646	SOAT1	-0.43	-0.71	-0.42	-0.74
5411	PNN	-0.81	-0.73	-0.37	-0.39
890	CCNA2	-1.64	-2.09	-1.21	-1.09
891	CCNB1	-1.67	-2.03	-1.12	-1.44
993	CDC25A	-1.56	-2.28	-2.34	-1.25
3621	ING1	0.03	0.50	0.20	0.13
5420	PODXL	1.54	0.54	0.80	0.66

ENTREZ GENE_ID	TargetID	Fold change relative to H441 (log2)			
		#12	#17	#40	#50
p21-induced genes					
5054	SERPINE1	1.87	0.28	1.27	1.69
5327	PLAT	1.72	0.43	2.47	0.97
3913	LAMB2	2.46	2.50	2.05	2.42
1824	DSC2	-1.30	-0.10	-0.70	-1.09
3958	LGALS3	0.05	1.01	-0.56	0.47
3959	LGALS3BP	0.18	-0.19	0.65	-0.21
5660	PSAP	0.60	0.99	0.86	0.52
1490	CTGF	5.57	4.79	5.42	4.86
2896	GRN	0.67	1.22	0.63	0.82
4820	NKTR	0.75	0.79	0.87	0.66
7052	TGM2	0.57	-0.31	-0.38	-0.03
10234	LRRC17	2.75	2.48	3.06	2.28
6289	SAA2	-0.18	1.06	-0.71	2.42
351	APP	0.64	-0.06	1.53	1.16
718	C3	0.57	0.61	-0.29	0.77
6695	SPOCK1	4.07	4.65	4.33	3.85
3690	ITGB3	0.11	0.06	0.98	0.03
2588	GALNS	-0.11	0.36	0.11	0.03
2548	GAA	0.45	-0.52	-1.36	0.14
3988	LIPA	0.67	0.41	0.91	0.22
1200	TPP1	1.93	1.93	2.35	2.17
6648	SOD2	0.45	2.21	0.22	2.19
2630	GBAP	0.06	0.07	0.08	0.04
1666	DECR1	0.65	1.32	0.43	0.32
9246	UBE2L6	0.51	1.16	0.02	1.00
9101	USP8	-0.09	0.03	0.07	-0.25
10397	NDRG1	-0.96	-0.89	-0.57	-0.85
27063	ANKRD1	5.22	3.79	3.41	4.12
9961	MVP	3.02	2.48	3.17	2.98
10628	TXNIP	0.72	2.29	0.10	1.32
6237	RRAS	1.69	2.87	2.55	2.72
5872	RAB13	0.26	0.44	-0.33	0.57
6464	SHC1	1.92	0.79	1.47	0.77
27250	PDCD4	0.76	0.28	0.27	0.05
9235	IL32	2.06	3.59	1.61	3.32

ENTREZ GENE_ID	TargetID	Fold change relative to H441 (log2)			
		#12	#17	#40	#50
7294	TXK	0.75	0.23	0.55	0.67
6567	SLC16A2	1.04	1.77	1.44	0.98
10133	OPTN	1.51	2.69	2.56	2.55
1774	DNASE1L1	2.46	2.03	1.57	1.67
4779	NFE2L1	0.98	0.39	0.11	0.46
8337	HIST2H2AA3	0.77	1.74	-0.78	1.15
8349	HIST2H2BE	1.66	2.02	2.17	1.47
51651	PTRH2	-0.11	-0.92	-1.61	-0.26

The list of p21-regulated genes was identified by Chang et al., 2000.

SUPPLEMENTARY REFERENCES

1. Chang BD, Watanabe K, Broude EV, Fang J, Poole JC, Kalinichenko TV and Roninson IB. Effects of p21Waf1/Cip1/Sdi1 on cellular gene expression: implications for carcinogenesis, senescence, and age-related diseases. Proc Natl Acad Sci U S A. 2000; 97(8):4291–4296.
2. Bhattacharjee A, Richards WG, Staunton J, Li C, Monti S, Vasa P, Ladd C, Beheshti J, Bueno R, Gillette M, Loda M, Weber G, Mark EJ, Lander ES, Wong W and Johnson BE. Classification of human lung carcinomas by mRNA expression profiling reveals distinct adenocarcinoma subclasses. Proc Natl Acad Sci U S A. 2001; 98(24): 13790–13795.
3. Wachi S, Yoneda K and Wu R. Interactome-transcriptome analysis reveals the high centrality of genes differentially expressed in lung cancer tissues. Bioinformatics. 2005; 21(23):4205–4208.
4. Beer DG, Kardia SL, Huang CC, Giordano TJ, Levin AM, Misek DE, Lin L, Chen G, Gharib TG, Thomas DG, Lizyness ML, Kuick R, Hayasaka S, Taylor JM, Iannettoni MD and Orringer MB. Gene-expression profiles predict survival of patients with lung adenocarcinoma. Nat Med. 2002; 8(8):816–824.
5. Hou J, Aerts J, den Hamer B, van Ijcken W, den Bakker M, Riegman P, van der Leest C, van der Spek P, Foekens JA, Hoogsteden HC, Grosveld F and Philipsen S. Gene expression-based classification of non-small cell lung carcinomas and survival prediction. PLoS One. 2010; 5(4):e10312.
